# Effects of Iranian herbal Zofa^®^ syrup for the management of clinical symptoms in patients with COVID-19: A randomized clinical trial

**DOI:** 10.22038/AJP.2023.21909

**Published:** 2023

**Authors:** Ali Ghazvini, Amir Vahedian-azimi, Morteza Abdoli, Farshid Rahimibashar, Yunes Panahi, Thozhukat Sathyapalan, Amirhossein Sahebkar

**Affiliations:** 1 *Chemical Injuries Research Center, Systems Biology and Poisonings Institute, Baqiyatallah University of Medical Sciences, Tehran, Iran*; 2 *Trauma Research Center, Nursing Faculty, Baqiyatallah University of Medical Sciences, Tehran, Iran *; 3 *Department of Anesthesiology and Critical Care, School of Medicine, Hamadan University of Medical Sciences, Hamadan, Iran *; 4 *Pharmacotherapy Department, Faculty of Pharmacy, Baqiyatallah University of Medical Sciences,* *Tehran, Iran *; 5 *Academic Diabetes, Endocrinology and Metabolism, Allam Diabetes Centre Hull Royal Infirmary Anlaby Road HU3 2JZ, Hull, UK*; 6 *Applied Biomedical Research Center, Mashhad University of Medical Sciences, Tehran, Iran*; 7 *Biotechnology Research Center, Pharmaceutical Technology Institute, Mashhad University of Medical Sciences, Tehran, Iran*

**Keywords:** Coronavirus disease 2019, Herbal medicine, Respiratory infection, Symptoms, Iran

## Abstract

**Objective:**

The objective of this study was to determine the role of Iranian herbal Zofa^®^ syrup in improving the clinical symptoms of patients with COVID-19.

**Materials and Methods:**

This randomized clinical trial was conducted on 105 patients with COVID-19. Patients were randomly assigned to the intervention (n=35) group (received 10 ml of Zofa^®^ syrup every 8 hours/seven days plus standard treatment) or the control (n=70) group (received only standard treatment). Assessments were performed before and after treatment.

**Results:**

The groups were comparable regarding age (p=0.980), gender (p=0.584), comorbidities (p=0.318), or drug history (p=0.771). There was no difference between patients' recovery status at the time of discharge (p=0.327) or two weeks post-discharge (p=0.165) in the intervention and control groups. No patient was hospitalized to the intensive care unit (ICU) for supplemental oxygen therapy and no patient died in the intervention group. However, in the control group, three (4.5%) patients were transferred to the ICU, and two (3.03%) patients died.

**Conclusion:**

Considering the better recovery status of the patients at the time of discharge and the absence of patient deaths in the intervention group, more additional studies are needed to confirm these findings and elucidate the role of Zofa^®^ in COVID-19.

## Introduction

The severe acute respiratory syndrome coronavirus 2 (SARS-CoV-2) was emerged in late 2019 in China; more than 274 million infected patients have been recorded globally, and more than 5 million lives have been lost. Although COVID-19 pathogenesis is still being investigated, changes in the immune response in the host appear has an important role (Bohn et al., 2020 ▶). Viral infection and replication can lead to increased levels of proinflammatory mediators, also known as cytokine storm (Costela-Ruiz et al., 2020 ▶). This can lead to acute inflammation, hyperimmune response, coagulopathy, and thromboembolic sequelae which can have detrimental effects on these patients (Bhattacharyya et al., 2020 ▶).

Mild symptoms of COVID-19 are cough (mostly dry cough, but in some cases productive cough), sore throat, headache, fatigue, fever, diarrhea, and anosmia. Severe COVID-19 is related to dyspnea, chest pain, confusion, and anorexia which may lead to acute respiratory distress syndrome (ARDS), and organ failure, as well as death. The only reported symptom that occurs most frequently in nearly 70% of patients is dry cough (Song et al., 2021 ▶). Although coughing is a protective reflex of the respiratory system, excessive cough can cause a variety of complications including headache, laryngeal trauma, throat pain and scratching, difficulty in swallowing, and brady- or tachyarrhythmias (Irwin et al., 2020 ▶; Jacobs et al., 2020 ▶). A persistent cough may reduce life satisfaction by interfering with sleep and normal activities. Coughing may continue for several months after recovery from COVID-19, leading to substantial community morbidity (Perotin et al., 2018 ▶). Coughing is also one of the most common ways for viruses to spread (Wiersinga et al., 2020 ▶). Hence, this COVID -19 associated symptom would necessitate optimal public health management. 

Although more than 45% of the global population is now fully vaccinated, this is insufficient to stop the virus's further worldwide spread. Multiple variants of SARS-CoV-2 with new features which affect virus properties such as transmissibility, as well as virulence have been discovered, making efforts to end the pandemic even more difficult. In addition, some of these variants may even circumvent the vaccine's protective effect (Harvey et al., 2021 ▶; Otto et al., 2021 ▶). The virus can be transmitted by SARS-CoV-2 carriers and chronic complications have emerged from "long COVID" disease (Proal and VanElzakker, 2021 ▶). As long as more than 90% of the world's population is not vaccinated against COVID-19, effective treatments are still needed to minimize viral spread. Traditional herbal remedies as adjunctive therapies for COVID-19 can be good choices as they have been studied in various countries (Ahmadi et al., 2021 ▶; Pawar et al., 2021 ▶; Yang et al., 2020 ▶). Zofa^®^ syrup is a herbal combination of traditional Iranian medicine used to treat respiratory diseases and contains components of several plants. This syrup is prescribed in the case of dry and allergic cough. It can also be used to treat the sore throat and common cold. In addition, it helps to reduce and prevention of asthma attacks and inflammation of the respiratory system. In this regard, this trial investigated the effectiveness of Zofa^®^ syrup as a complementary therapy for managing clinical symptoms in patients with COVID-19.

## Materials and Methods

**Trial design and ethical approval**

This prospective, single-center, randomized clinical trial was conducted from April 2020 until May 2020 to determine the efficacy of Zofa^®^ syrup in clinical symptoms management of patients infected with COVID-19 admitted to Baqiyatallah hospital, Tehran, Iran. The Consolidated Standards of Reporting Trials (CONSORT) statement was used to review all aspects of the study (Cuschieri, 2019). The Ethics Committee of Baqiyatallah University of Medical Sciences reviewed and approved protocol of the study (IR.BMSU.REC.1399.027). This trial was also registered in the Iranian registry of clinical trials (IRCT20080901001165N48). Each individual filled a written informed consent. The study was performed based on the Declaration of Helsinki (Helsinki, 2013 ▶). 

**Study population**

We enrolled symptomatic adult patients (over the age of 18) with positive COVID-19 diagnosis based on a positive real-time polymerase chain reaction (RT-PCR) of the respiratory tract samples and a chest computed tomography (CT). The followings were the exclusion criteria: (a) admission to the intensive care unit (ICU) because of intubation or infection exacerbation or severe adverse drug reactions (ADRs) occurred, (b) patients who had symptoms for more than seven days, (c) patients who were simultaneously in a clinical trial, or (d) patients who refused to participate in the study. All included patients were randomly assigned into control or intervention groups. In the control group, patients received standard treatment according to the last national and international guideline for the recommended treatment-based Ministry of Health of Iran protocols of COVID-19 (Rahmanzade et al., 2020 ▶), and the World Health Organization (WHO) (Schoen et al., 2019). However, in addition to standard treatment, patients in the intervention group received 10 ml of Zofa^®^ syrup every 8 hr. for seven days. 

**Randomization**

Using the Block randomization method, patients in the intervention group were administered Zofa^®^ syrup plus standard treatment while the control group only received the standard treatment. A computer-generated randomization code was applied in permuted blocks of six. Random Allocation Software © (RAS; Informer Technologies, Inc., Madrid, Spain) performed block randomization using a sealed envelope technique and computer-generated random numbers.

**Study medication**

Niak Pharmaceutical Company manufactured Zofa^®^ syrup in Tehran, Iran (http://www.niakpharma.com/index.php/en/2019-03-08-09-11-07/2019-03-08-09-18-54/38-zofa-syrup). Zofa^®^ syrup is a polyherbal drug consisting of *Hyssopus officinalis* (hyssop), Morning glory, *Echium vulgare* (viper’s bugloss), *Ziziphus jujuba* (jujube), Zante Currant, Fig, Cordia fruit, Quince, *Glycyrrhiza glabra* (liquorice), Maidenhair, Caper, Marshmallow, Melon seed, Cucumber seed, Nut mace, sweet violet, Acacia Gum, Mallow, Tragacanth Gum, Stevia and Honey. 

**Data collection**

Patient's demographic data (age and sex), high-risk behavior (contact with an infected person or recently travelling), drug history (immunosuppressive, corticosteroids, and cardiovascular drugs) and comorbidities (myocardial infarction [MI], diabetes mellitus [DM], ischemic heart disease [IHD], hypertension [HTN], rheumatology, congestive heart failure [CHF], asthma, chronic obstructive pulmonary disease [COPD] and malignancy) were recorded at the beginning of the trial by the physician. Furthermore, the pulmonologist assessed patients based on the various symptoms and signs of COVID-19 infection (fever, dry cough, productive cough, body pain, chest pain, shortness of breath, anorexia, fatigue, sore throat, headache, chill, loss of taste/smell, nausea/vomiting, and diarrhea) and vital signs (saturated pressure of oxygen [SPO2], heart rate [HR], blood pressure [BP] body temperature and respiratory rate [RR]). All symptoms and vital signs were collected for each patient before and after the intervention. Hospitalization, admission, and discharge dates were recorded for each patient and the patients were followed up 14 days after discharge from the hospital and all clinical symptoms and mortality were recorded in them.

Venous blood was dispensed into 5 ml SST tubes and 2 ml K_3_EDTA to gel for biochemistry and hematology analysis, respectively. Within eight hours of the blood draw, a hematological measurement (complete blood count (CBC) test with 3-part differential) was performed. The three-part differential hematological parameters, including hemoglobin (Hb), red blood cell (RBC), count, lymphocyte count, platelet count (PLTs) and white blood cells (WBC), were recorded for each individual. The serum was used for the analyses of biochemical parameters, such as creatinine, phosphokinase (CPK), creatinine (Cr), C-reactive protein (CRP), procalcitonin (PCT), blood urea nitrogen (BUN), sodium (NA) and potassium (K).

**Chest CT scan**

Chest CT-scan was performed for all patients in a supine position before and after the intervention. All CT scans were performed using 16-row detector CT scanner (general electric, GE, optima, USA). Two highly experienced radiologists in thoracic imaging reviewed all chest CTs. The chest CT scan was assessed according to the Fleischner Society Nomenclature recommendations (Hansell et al., 2008). In addition, CT images were assessed for the lesion types (clear ground glass opacity [GGO], consolidation, crazy paving, and combination of GGO + consolidation) and lesions’ locations (unilateral and bilateral). 

**Statistical analysis**

SPSS software (ver.21) (SPSS Inc., IL, and Chicago, USA) was used for statistical analysis. The Kolmogorov-Smirnov test was performed to determine if the numeric variables followed a normal distribution. For the Numeric Normal, data are presented as mean ± standard deviation (SD) and frequency (percent) for categorical variables. The chi-square or Fisher’s exact test was performed for categorical data. A t-test or Mann-Whitney U test was selected to analyze the continuous data. Time main effect based on repeated measures ANOVA “RMANOVA” to compare the mean parameters in pre- and post-intervention within the group was used. Analysis of covariance “ANCOVA” was used for post-intervention values and was adjusted for age, comorbidities, drug history, and gender. In addition, the time-by intervention interaction effect based on RMANOVA was calculated. Mauchly's sphericity test addressed the assumption of sphericity, and in case of dissatisfaction, the Greenhouse-Geiser correction of the p-value was utilized. In all analyses, p-values less than 0.05 were considered significant.

## Results

**Trial population**

**Figure 1:**
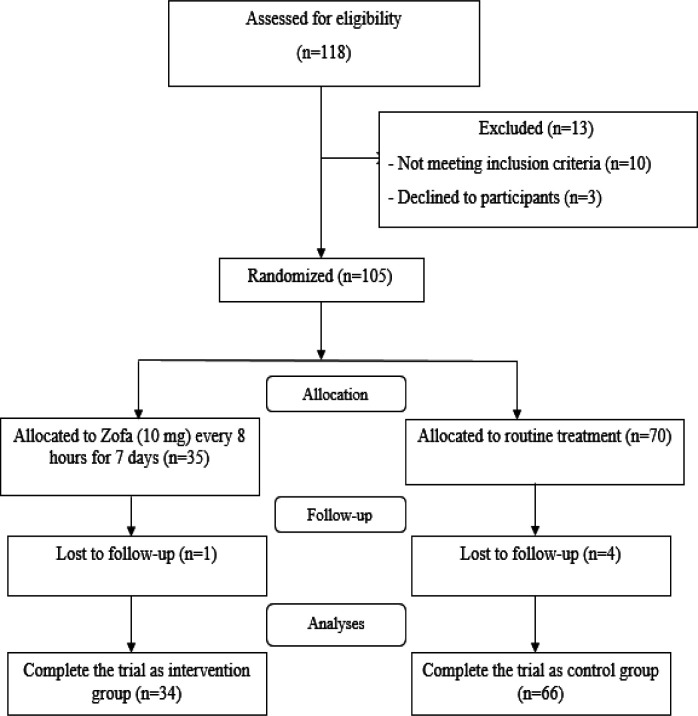
CONSORT flow diagram for this study

From April 2020 to May 2020, 105 out of 118 suspected COVID-19 patients who were admitted to Baqiyatallah Hospital in Tehran, Iran, were studied. These individuals had fulfilled the inclusion criteria and were willing to participate in the study. Four patients in the control group and one in the intervention group were excluded during the follow-up due to the infection exacerbation. Finally, of the 100 eligible patients with COVID-19, 34 and 66 patients remained in the intervention and control groups, respectively. Figure 1 demonstrates the participants’ allocation to the RCT based on the consolidated standards of reporting trials (CONSORT).

**Baseline demographic characteristics and clinical status**

The demographic characteristics and clinical status of the participants in each group are presented in Table 1. There were 21 (61.8%) male participants in the intervention and 37 (56.1%) in the control group (p=0.584). The mean age of the intervention and control groups was 55.09±16.63 and 55.02±12.66 years, respectively (p=0.980). There was no statistical difference between the two groups in terms of age (p=0.980), gender (p=0.584), comorbidities (p=0.318), or drug history (p=0.771). Only five patients reported high-risk behavior, of which, 2 (3.03%) patients in the control group and 1 (2.9%) patient in the intervention group had contact with an infected person, and only 2 (3.03%) patients in the control group had recently travelled. Four common symptoms among both trial arms were shortness of breath (65%), dry cough (56%), fever (56%), and sore throat (39%), respectively. There was no significant difference in the frequency of symptoms before intervention between the Zofa^®^ and placebo groups (p>0.05). 

**Table 1:** Comparison of baseline demographic and clinical characteristics between the two groups of the study

**Variables**		**Intervention group (n=34)**	**Control group (n=66)**	**p-value**
**Age **(year)	Mean ± SD	55.09±16.63	55.02±12.66	0.980
	Median (IQR)	54 (44-65)	54 (48-63)	
	(Range)	(26-92)	(18-90)	
**Gender**	Male (%)	21 (61.8)	37 (56.1)	0.584
	Female (%)	13 (38.2)	29 (43.9)	
**Drug history**	Yes (%)	7 (20.6)	12 (18.2)	0.771
	No (%)	27 (79.4)	54 (81.8)	
Table 1. Continue				
**Drug history types**	Immunosuppressive (%)	1 (2.9)	1 (1.5)	1
	Corticosteroids (%)	1 (2.9)	0	-
	Cardiovascular drugs (%)	6 (17.6)	12 (18.2)	0.947
**Comorbidities**	Yes (%)	16 (47.1)	38 (57.6)	0.318
	No (%)	18 (52.9)	28 (42.4)	
**Comorbidity types**	MI (%)	1 (2.9)	0	-
	IHD (%)	4 (11.8)	9 (13.6)	0.792
	DM (%)	5 (14.7)	20 (30.3)	0.088
	HTN (%)	6 (17.6)	14 (21.2)	0.673
	COPD (%)	1 (2.9)	0	-
	Rheumatology (%)	0	2 (3)	-
	Asthma (%)	2 (5.9)	1 (1.5)	0.266
	Malignancy (%)	0	2 (3)	-
	Others (%)	8 (23.5)	18 (27.3)	0.686
**Symptoms**	Fever (%)	17 (50)	39 (59.1)	0.386
	Productive cough (%)	5 (14.2)	14 (21.2)	0.432
	Dry cough (%)	19 (55.9)	37 (56.1)	0.986
	Shortness of breath (%)	23 (67.6)	42 (63.3)	0.690
	Body pain (%)	14 (41.2)	20 (30.3)	0.277
	Chest pain (%)	8 (23.5)	8 (12.1)	0.069
	Anorexia (%)	9 (26.5)	20 (30.8)	0.655
	Fatigue (%)	10 (29.4)	21 (31.8)	0.805
	Lost sense of smell/taste (%)	4 (11.8)	3 (4.5)	0.224
	Nausea/vomiting (%)	10 (29.4)	11 (16.7)	0.138
	Diarrhea (%)	3 (8.8)	8 (12.1)	0.745
	Constipation (%)	10 (29.4)	14 (21.2)	0.654
	Chill (%)	6 (17.6)	15 (22.7)	0.555
	Sore throat (%)	12 (35.3)	27 (40.9)	0.586
	Other symptoms (%)	3 (8.8)	6 (9.1)	0.999

Abbreviations; Myocardial infarction (MI), Ischemic heart disease (IHD), Diabetes mellitus (DM), Hypertension (HTN), and Chronic obstructive pulmonary disease (COPD)

**Chest CT findings**

CT image findings between the two groups prior to and after the intervention are presented in Table 2. Initial chest CT scans (pre-intervention) were performed on 68 out of 100 patients (44 in the control group and 24 in the intervention group), and one patient in the control group had clear lungs. The common chest CT features in all participants were mixed GGO and consolidation (35/67, 52.2%), pure GGO (30/67, 44.8%), and pure crazy-paving pattern (2/67, 3%). The incidences of pure GGO in the intervention group were significantly higher than in the control group (70.8% vs. 29.5%, p=0.019). However, control patients showed a higher incidence of mixed GGO and consolidation than the intervention group (68.2% vs. 20.8%, p<0.001). Initial chest CT scans showed bilateral involvement in most individuals (89.5%) of the participants, with 95.8% and 84.1% reported in the intervention and control groups, respectively. Post-intervention chest CT scans were performed on 34 out of 100 patients (27 in the control group and 7 in the intervention group). Following the treatment process, the lungs of two patients, one in the intervention group and the other in the control group, were cleared of the virus. There was no statistical difference between the two groups in terms of both lesion types (p=0.862) and lesion distribution (p=0.858).

**Table 2:** Comparison of chest CT findings on pre- and post-intervention between the intervention and control groups

**Time/ chest CT findings**		**Intervention group (n=24)**	**Control group (n=44)**	**p-value **
**Lesion types**				
**Pre-intervention**	Clear	0	1 (2.3)	
	GGO	17 (70.8)	13 (29.5)	
	Consolidation	0	0	
	Crazy paving	2 (8.3)	0	
	GGO + consolidation	5 (20.8)	30 (68.2)	
**Lesions distribution**				0.246
**Pre-intervention**	No	0	2 (4.5)	
	Unilateral	1 (4.2)	5 (11.3)	
	Bilateral	23 (95.8)	37 (84.1)	
**Time/ chest CT findings**		Intervention group (n=7)	Control group (n=27)	p-value
**Lesion types**				0.862
**Post-intervention**	Clear	1 (14.3)	2 (7.4)	
	GGO	5 (71.4)	18 (66.7)	
	Consolidation	0	1 (3.7)	
	GGO + consolidation	1 (14.3)	6 (22.2)	
**Lesions distribution**				0.858
**Post-intervention**	Unilateral	0	1 (3.7)	
	Bilateral	6 (85.7)	23 (85.2)	

GG0: Ground-glass opacities

**Vital signs, and hematological and** **biochemical parameters** **findings**

Tables 3 and 4 show the comparisons of biochemical parameters and vital signs/hematology before and after intervention in the control and intervention groups, respectively. There were no significant differences between the two groups, before the trial, regarding hematological and biochemical factors, indicating the homogeneity of the study participants (p>0.05). Respiration rate was the only vital sign that differed between the two groups before the intervention, so the respiratory rate (breaths per minute) was significantly higher in the intervention group (32.33±35.13 vs. 18.66±3.20 P=0.034). The results of ANCOVA for postintervention measures adjusted for age, gender, drug history, and comorbidities as the confounders showed statistical difference in WBC (9.98±6.06 vs. 6.71±2.39, p=0.012), lymphocyte (5.52±8.51 vs. 22.79±12.38, p<0.001), BUN (17.17±8.09 vs. 14.25±5.78, P=0.045) and sodium (145.30±44.14 vs. 139.83±3.85, p=0.048) in post-intervention between the trial arms. According to the results of ANOVA with a repeated measure to compare the mean parameters in pre- and post-intervention within the group, there was a significant time effect on SPO2, BP, Hb and PLT in both trial arms. Significant differences regarding the time effect were observed in RR (18.66±3.20 vs. 30.01±29.40, p=0.063) and lymphocyte (22.20±9.27 vs. 5.52±8.51, p<0.001) within the control groups and in HR (92.50±13.02 vs. 81.50±7.74, p=0.017) within the intervention groups. In addition, comparisons between the groups in regard to the time by intervention interaction effect based on RMANOVA showed significant differences for WBC (p<0.001) and lymphocytes (p<0.001) (Parameter changes pre- and post-intervention are available in Figures 1 to 15 in Supplementary file 1).

**Final outcomes**

Post-intervention symptoms and clinical outcomes of the participants in the study are presented in Table 5. According to results, the symptoms that remained in patients after treatment and at the time of discharge included dry cough, fatigue, sore throat, body pain, and shortness of breath, although it was not different between the trial arms except fatigue that was significantly higher in the intervention group (13% vs. 0, p=0.031). In addition, there was no significant difference between patients' recovery status at the time of discharge (p=0.327) and two weeks after discharge (p=0.165) in the intervention and control groups. Ultimately, no patient needed to be admitted to the ICU for supplemental oxygen therapy, and no patient died in the intervention group. However, in the control group, three (4.5%) patients were transferred to the ICU, and two (3.03%) patients died.

**Table 3:** Comparison of vital signs and hematological parameters on pre- and post-trial between the intervention and control groups

**Parameters**	**Groups**	**Pre-intervention**	**Post-intervention**	**p-value**		
				Time effect***	Interventi effect****	Interaction effect*****
**SPO2 ** (%)	Control	89.71±7.02	94.78±2.30	<0.001*	0.843	0.329
	Intervention	90.85±2.76	94.01±3.11	0.007*		
	**p-value	0.640	0.319			
**BP ** (mmHg)	Control	123.60±9.81	112.94±13.74	0.010*	0.574	0.400
	Intervention	130.53±12.40	113.97±13.29	0.005*		
	**p-value	0.080	0.862			
**HR ** (BPM)	Control	85.40±18.24	83.01±8.98	0.644	0.344	0.237
	Intervention	92.50±13.02	81.50±7.74	0.017*		
	**p-value	0.080	0.679			
**RR ** (breaths per minute)	Control	18.66±3.20	30.01±29.40	0.063*	0.516	0.118
	Intervention	32.33±35.13	27.83±25.56	0.306		
	**p-value	0.034*	0.870			
**Body** **Temperature**	Control	36.78±0.70	36.64±0.44	0.650	0.217	0.651
	Intervention	36.90±0.76	36.57±0.46	0.122		
	**p-value	0.104	0.730			
**WBC** (×10^3 ^) U/L	Control	7.012±3.51	9.98±6.06	0.680	0.012*	<0.001*
	Intervention	6.51±3.01	6.71±2.39	0.729		
	**p-value	0.201	0.019*			
**Lymphocyte** (%)	Control	22.20±9.27	5.52±8.51	<0.001*	<0.001*	<0.001*
	Intervention	20.30±10.51	22.79±12.38	0.358		
	**p-value	0.708	<0.001*			
**RBC** (×10^3 ^) U/L	Control	4.55±0.77	4.46±1.33	0.686	0.429	0.070
	Intervention	4.79±0.54	4.47±0.33	0.069		
	**p-value	0.135	0.877			
**Hb** (g/dL)	Control	13.78±1.86	12.47±1.70	0.007*	0.246	0.462
	Intervention	14.14±1.70	12.66±1.20	0.005*		
	**p-value	0.400	0.660			
**PLT** (×10^3 ^) U/L	Control	212.56±82.12	310.62±99.21	<0.001*	0.365	0.650
	Intervention	202.38±61.98	292.92±68.44	<0.001*		
	**p-value	0.530	0.432			

Data are expressed as the Mean ± standard deviation (SD), SPO2: saturated pressure of oxygen, BP: blood pressure, HR: heart rate, RR: respiratory rate, WBC: withe blood cell, RBC: red blood cell, Hb: hemoglobin, PLT: Platelet, *P<0.05 considered as significant, ** Independent t-test to compare the mean of parameters in pre- and post-intervention between two groups, *** Time main effect based on repeated measures ANOVA “RMANOVA” to compare the mean parameters in pre- and post-intervention within the group, **** Analysis of covariance “ANCOVA” for after (post) intervention measures adjusted for age, gender, comorbidities and drug history, ***** Time by intervention interaction effect based on RMANOVA

**Table 4:** Comparison of biochemical parameters on pre- and post-trial between the intervention and control groups

**Parameters**	**Groups**	**Pre-intervention**	**Post-intervention**	**p-value**		
				**Time** effect ***	**Intervention** effect****	**Interaction** effect*****
**PCT** **(ng/ml)**	Control	0.255±0.088	0.230±0.096	0.226	0.177	0.262
	Intervention	0.314±0.089	0.271±0.099	0.423		
	**p-value	0.391	0.760			
**CRP** **(mg/L)**	Control	48.09±53.28	18.60±29.71	0.113	0.055	0.071
	Intervention	43.30±37.32	19.10±16.86	0.110		
	**p-value	0.619	0.622			
**BUN** **(mg/dl)**	Control	13.82±5.21	17.17±8.09	0.388	0.045*	0.300
	Intervention	12.75±4.93	14.25±5.78	0.164		
	**p-value	0.236	0.071			
**Cr** **(mg/dl)**	Control	0.939±0.229	0.963±0.308	0.716	0.212	0.526
	Intervention	0.925±0.166	0.900±0.133	0.097		
	**p-value	0.390	0.952			
**NA** **(mEq/L)**	Control	136.51±3.65	145.30±44.14	0.222	0.048*	0.499
	Intervention	138.20±4.13	139.83±3.85	0.156		
	**p-value	0.620	0.062			
**K** **(mmol/L)**	Control	7.48±17.47	4.28±0.618	0.332	0.246	0.906
	Intervention	6.50±9.53	4.22±0.501	0.341		
	**p-value	0.897	0.705			

Data are expressed as the Mean±standard deviation (SD), PCT: procalcitonin, CRP: C-reactive protein, BUN: Blood urea nitrogen, Cr: Creatinine, NA: sodium, K: potassium, *p<0.05 considered as significant, **Independent t-test to compare the mean of parameters in pre- and post-intervention between two groups, ***Time main effect based on repeated measures ANOVA “RMANOVA” to compare the mean parameters in pre- and post-intervention within the group, ****Analysis of covariance “ANCOVA” for after (post) intervention measures adjusted for age, gender, comorbidities and drug history, *****Time by intervention interaction effect based on RMANOVA

**Table 5:** Symptoms and clinical outcomes of the participants in the two groups of the study

**Variables**		**Intervention group**	**Control group**	**p-value**
**Symptoms**	Shortness of breath (%)	9 (39.1)	12 (25)	0.222
	Dry cough (%)	6 (26.1)	11 (22.9)	0.770
	Sore throat (%)	2 (8.7)	0	0.102
	Body pain (%)	2 (8.7)	1 (2.1)	0.243
	Fatigue	3 (13)	0	0.031*
**Hospitalization days**	Mean ± SD	5.68±2.74	6.90±6.14	0.286
	Median (IQR)	5 (4-7)	5 (4-7)	
	(Range)	(1-12)	(1-40)	
**Days of study**	Mean ± SD	4.0±2.79	5.79±4.80	0.146
	Median (IQR)	4 (1-6)	4 (2-8)	
	(Range)	(1-10)	(1-17)	
**Discharge status**	With symptoms (%)	12 (35.3)	31 (47)	0.327
	Without symptoms (%)	12 (35.3)	19 (28.8)	
**Status of patients after ** **two weeks of hospital** **discharge**	Good (%)	17 (50)	41 (62.1)	0.165
	With symptoms (%)	6 (17.6)	6 (9.1)	
	Death	0	2 (3.03)	

*p<0.05 considered significant

## Discussion

In this clinical trial study, the effect of Iranian Zofa^®^ syrup on the clinical outcomes of patients with COVID-19 was investigated. This syrup is a combination of several plants used in Iranian traditional medicine (ITM) for respiratory infections. 

During the 7 days of treatment and the 14 days of follow-up of patients after discharge from the hospital, many symptoms such as fever, productive cough, chest pain, anorexia, headache, nausea/vomiting, diarrhea, constipation and loss of smell/gradually improved in both groups. However, the symptoms such as dry cough, fatigue, sore throat, body pain, and shortness of breath were reported to be lessened compared with baseline, although they were not different between the trial arms. In the intervention group, no patient needed to be admitted to the ICU for supplemental oxygen therapy, and no patient died. However, in the control group, three patients were transferred to the ICU, and two patients died. Vital signs, including oxygen saturation and heart rate, followed a steady pattern during the follow-up and remained similar between the two groups.

While the advent of effective vaccines has given the government, the scientific community, and the general public hope for an end to the pandemic, there is still a long way to go before global vaccination is complete. In addition, the appearance of different variants of the virus makes efforts to end the pandemic more difficult, so some of these variants may even be able to circumvent the protective effect of the vaccine. Therefore, traditional and herbal medicine approaches could provide helpful interventions in this regard (Babich et al., 2020 ▶; Paudyal et al., 2021 ▶; Hosseini et al., 2021 ▶; Zahedipour et al., 2020 ▶; Vahedian-Azimi et al., 2022 ▶). Given this potential, traditional medicine offers researchers potential drugs that have successfully been applied in other diseases. Herbal medications are shown to have the potential to improve viral infections of the respiratory system (Fiore et al., 2008 ▶; Monavari et al., 2007 ▶; Karsch-Völk et al., 2014 ▶). Currently, there is no data on the effects of Zofa^®^ syrup made by Niak Pharmaceuticals on people suffering from SARS-CoV-2. However, the effectiveness of Zofa^®^ syrup compounds such as Hyssop for respiratory diseases has been investigated in previous studies (Iranzadasl et al., 2021 ▶; Choopani et al., 2015 ▶). The Hyssop plant (*Hyssopus officinalis *L.) is mostly found in the Middle East, Southern Europe, and near Caspian Sea in Iran. It is largely recognized in ITM for the treatment of cold catarrh, cough, pneumonia, and asthma, as well as other inflammatory pulmonary disorders (Vlase et al., 2014 ▶; Sokmen et al., 2004 ▶; Süleyman et al., 2010 ▶). Hyssop extracts are enriched with numerous compounds which enhance its health-preserving properties (Borrelli et al., 2019 ▶). The chemical and biological properties of *Hyssopus officinalis* L. leaf extracts and essential oils have been extensively researched. Its antimicrobial, insecticidal, antioxidant, antiviral, and antifungal impacts have been studied. This plant has antiviral activity since it contains tannins, and caffeic acid as well as unidentified high-molecular compounds (Pandey et al., 2014 ▶; Letessier et al., 2001 ▶).

In addition, other components of Zofa^®^ potentially affect the treatment of respiratory infections. Nut mace extracts obtained from the wood of *Myristica fragrans Houtt* are known as traditional anti-allergy, anti-pyretic, and anti-inflammatory remedies due to the presence of alpha-pinene (Champasuri and Itharat, 2016 ▶). The medicinal plant marshmallow *Althaea officinalis *L.* (A. officinalis)*, has been used to treat cough for centuries (Bonaterra et al., 2020 ▶; Banaee et al., 2017 ▶). Marshmallows and mallow are an expectorant, and because of the high mucilage content, they have a soothing effect on respiratory tracts. *Ziziphus jujuba* is a member of the family Rhamnaceae, which is widespread in tropical and subtropical countries (Banaee et al., 2017 ▶; Martins et al., 2017 ▶). It has traditionally been used for pharmacological goals including anti-inflammation, antidiarrheal and antibacterial, vasopressor, and sedative which could be due to the presence of betulinic acid and quercetin (Rajaei et al., 2021 ▶; Mesaik et al., 2018 ▶). Due to the role of medicinal herbs in Zofa^®^ compounds in the management of respiratory infections, this Iranian syrup may be able to minimize the clinical symptoms of COVID-19. However, further studies are needed to confirm these findings and elucidate Zofa^®^'s role in COVID-19 treatment.

The main advantage of this study was the careful follow-up and patient monitoring so that all patients were hospitalized, and the treatment was the same in both groups except for Zofa^®^ syrup. We followed the approved COVID-19 treatment protocols of Ministry of Health of Iran and WHO (Rahmanzade et al., 2020 ▶; Schoen et al., 2019 ▶). Regarding the limitations of the study, we used the minimum but effective drug dosage to avoid complications. Second, repeating and conducting some important laboratory indices was not possible for all patients considering the unusual circumstances of the pandemic.

We investigated the effects of Zofa^®^ syrup, an herbal drug for the treatment of respiratory symptoms, in patients with COVID-19. According to our results, the patients' recovery status at the time of discharge and two weeks after discharge was better in the intervention group, and not even one mortality was observed in this group. But these differences were not statistically significant. Therefore, further studies appear to be needed to confirm these findings and elucidate the role of Zofa^®^ in COVID-19.

## Conflicts of interest

The authors have declared that there is no conflict of interest.
